# [2,9-Bis(3,5-dimethyl-1*H*-pyrazol-1-yl-κ*N* ^2^)-1,10-phenanthroline-κ^2^ *N*,*N*′]bis­(thio­cyanato-κ*N*)cadmium(II)

**DOI:** 10.1107/S1600536810051275

**Published:** 2010-12-11

**Authors:** Lu Yi Zheng, Yan Hui Chi

**Affiliations:** a College of Chemistry and Chemical Engineering, University of Jinan, Jinan 250022, People’s Republic of China; b Department of Chemistry, Shandong Normal University, Jinan 250014, People’s Republic of China

## Abstract

In the title complex, [Cd(NCS)_2_(C_22_H_20_N_6_)], the Cd^II^ ion is in a CdN_6_ coordination geometry which is inter­mediate between octa­hedral and trigonal–prismatic. The dihedral angles formed between the mean planes of the pyrazole rings and the phenanthroline system are 15.74 (15) and 16.30 (13)°. In the crystal, there is a π–π stacking inter­action involving two symmetry-related pyrazole rings, with a centroid–centroid distance of 3.664 (3) Å. In addition, there is a relatively short inter­molecular contact between C atoms [C⋯C = 3.399 (6) Å] involving symmetry-related pyridine rings along the *a* axis.

## Related literature

For a related structure, see: Wang *et al.* (2009 ▶).
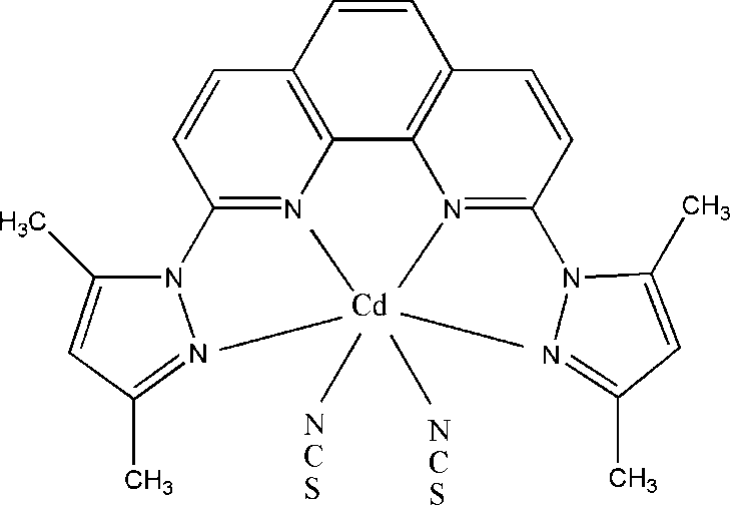


## Experimental

### 

#### Crystal data

[Cd(NCS)_2_(C_22_H_20_N_6_)]
*M* *_r_* = 597.00
Monoclinic, 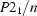

*a* = 8.1350 (15) Å
*b* = 20.601 (4) Å
*c* = 14.633 (3) Å
β = 99.323 (3)°
*V* = 2420.0 (8) Å^3^
*Z* = 4
Mo *K*α radiation
μ = 1.11 mm^−1^
*T* = 298 K
0.35 × 0.10 × 0.08 mm

#### Data collection

Bruker SMART APEX CCD diffractometer
Absorption correction: multi-scan (*SADABS*; Sheldrick, 1996 ▶) *T*_min_ = 0.698, *T*_max_ = 0.917
14038 measured reflections
5272 independent reflections
4099 reflections with *I* > 2σ(*I*)
*R*_int_ = 0.042

#### Refinement

*R*[*F* ^2^ > 2σ(*F* ^2^)] = 0.046
*wR*(*F* ^2^) = 0.101
*S* = 1.05
5272 reflections
320 parameters
H-atom parameters constrained
Δρ_max_ = 0.69 e Å^−3^
Δρ_min_ = −0.56 e Å^−3^

### 

Data collection: *SMART* (Bruker, 1997 ▶); cell refinement: *SAINT* (Bruker, 1997 ▶); data reduction: *SAINT*; program(s) used to solve structure: *SHELXTL* (Sheldrick, 2008 ▶); program(s) used to refine structure: *SHELXTL*; molecular graphics: *SHELXTL*; software used to prepare material for publication: *SHELXTL*.

## Supplementary Material

Crystal structure: contains datablocks I, global. DOI: 10.1107/S1600536810051275/lh5181sup1.cif

Structure factors: contains datablocks I. DOI: 10.1107/S1600536810051275/lh5181Isup2.hkl

Additional supplementary materials:  crystallographic information; 3D view; checkCIF report

## Figures and Tables

**Fig. 1.**
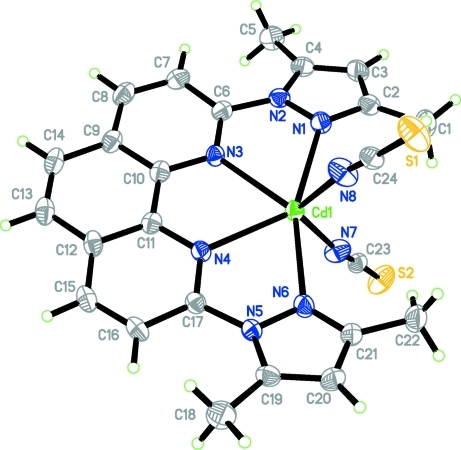
The molecular structure of title compound with the atom-numbering scheme. Displacement ellipsoids are drawn at the 30% probability level.

**Fig. 2.**
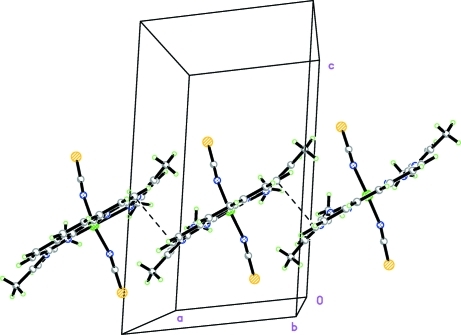
Part of the crystal structure with short C···C contacts drawn as dashed lines.

## supplementary crystallographic information

### Comment

Derivatives of 1,10-phenanthroline play an important role in modern coordination chemistry and many complexes have been reported with these types of compounds as ligands [see e.g. Wang et al. (2009) for a closely related Cd complex]. To the best of our knowledge, no crystal structures of complexes with 2,9-bis(3,5-Dimethyl-1H-pyrazol-1-yl)-1,10-phenanthroline as a ligand have been reported so far. Herein we report the crystal structure of the title compound (I).

Fig. 1 shows the title complex. The Cd^II^ ion is in a CdN_6_ coordination geometry which is approximately intermediate between octahedral and triginal-prismatic and this may be attributed to the chelation mode of the 2,9-bis(3,5-dimethyl-1H-pyrazol-1-yl)-1,10-phenanthroline ligand. The dihedral angles between the planes that consist of the non-hydrogen atoms of the 1,10-phenanthroline ring system and the pyrazole rings are 15.74 (15)° (involving the pyrazole ring containing atoms N1 and N2) and 16.30 (13)° (involving the pyrazole ring containing atoms N5 and N6), respectively. In the crystal structure, there is a π–π stacking interaction involving symmetry related pyrazole rings, with the relevant distance being Cg1···Cg2^i^ 3.664 (3) Å and Cg1···Cg2^i^_perp_ = 3.610 Å (symmetry code: (i) 1+x, y, z; Cg1 and Cg2 are the centroids of C2-C4/N1N2 pyrazole ring and C19-C21/N5N6 pyrazole ring, respectively; Cg1···Cg2^i^_perp_ is the perpendicular distance from Cg1 ring to Cg2^i^ ring). In addition, there is a relatively short intermolecular contact between atom C16 and C7^ii^ (symmetry code: (ii) -1+x, y, z with a C···C separation of 3.399 (6) Å involving pyridine rings along the a axis (Fig. 2).

### Experimental

A 10 ml methanol solution of Cd(ClO_4_).6H_2_O (0.0744 g, 0.177 mmol) was added into 10 ml dichloromethane solution of 2,9-bis(3,5-Dimethyl-1*H*-pyrazol-1-yl)-1,10-phenanthroline (0.0299 g, 0.081 mmol) in drops, and 5 ml of methanol solution containing NaNCS (0.0149 g, 0.184 mmol) was added into the mixed soluton.  This solution was stirred for a few minutes. Colorless single crystals were obtained after the filtrate had been allowed to stand at room temperature for about a week.

### Refinement

All H atoms were placed in calculated positions and refined as riding with C—H = 0.96 Å, *U*_iso_ = 1.5*U*_eq_(C) for methyl H and C—H = 0.93 Å, *U*_iso_ = 1.2*U*_eq_(C) for other H atoms.

### Crystal data

**Table:**

[Cd(NCS)_2_(C_22_H_20_N_6_)]	*F*(000) = 1200
*M**_r_* = 597.00	*D*_x_ = 1.639 Mg m^−^^3^
Monoclinic, *P*2_1_/*n*	Mo *K*α radiation, λ = 0.71073 Å
Hall symbol: -P 2yn	Cell parameters from 3140 reflections
*a* = 8.1350 (15) Å	θ = 2.4–23.4°
*b* = 20.601 (4) Å	µ = 1.11 mm^−^^1^
*c* = 14.633 (3) Å	*T* = 298 K
β = 99.323 (3)°	Block, colorless
*V* = 2420.0 (8) Å^3^	0.35 × 0.10 × 0.08 mm
*Z* = 4	

### Data collection

**Table:**

Bruker SMART APEX CCD diffractometer	5272 independent reflections
Radiation source: fine-focus sealed tube	4099 reflections with *I* > 2σ(*I*)
graphite	*R*_int_ = 0.042
φ and ω scans	θ_max_ = 27.0°, θ_min_ = 1.7°
Absorption correction: multi-scan (*SADABS*; Sheldrick, 1996)	*h* = −10→8
*T*_min_ = 0.698, *T*_max_ = 0.917	*k* = −26→26
14038 measured reflections	*l* = −15→18

### Refinement

**Table:**

Refinement on *F*^2^	Primary atom site location: structure-invariant direct methods
Least-squares matrix: full	Secondary atom site location: difference Fourier map
*R*[*F*^2^ > 2σ(*F*^2^)] = 0.046	Hydrogen site location: inferred from neighbouring sites
*wR*(*F*^2^) = 0.101	H-atom parameters constrained
*S* = 1.05	*w* = 1/[σ^2^(*F*_o_^2^) + (0.045*P*)^2^] where *P* = (*F*_o_^2^ + 2*F*_c_^2^)/3
5272 reflections	(Δ/σ)_max_ = 0.008
320 parameters	Δρ_max_ = 0.69 e Å^−^^3^
0 restraints	Δρ_min_ = −0.56 e Å^−^^3^

### Special details

**Table:**

Geometry. All e.s.d.'s (except the e.s.d. in the dihedral angle between two l.s. planes) are estimated using the full covariance matrix. The cell e.s.d.'s are taken into account individually in the estimation of e.s.d.'s in distances, angles and torsion angles; correlations between e.s.d.'s in cell parameters are only used when they are defined by crystal symmetry. An approximate (isotropic) treatment of cell e.s.d.'s is used for estimating e.s.d.'s involving l.s. planes.
Refinement. Refinement of *F*^2^ against ALL reflections. The weighted *R*-factor *wR* and goodness of fit *S* are based on *F*^2^, conventional *R*-factors *R* are based on *F*, with *F* set to zero for negative *F*^2^. The threshold expression of *F*^2^ > σ(*F*^2^) is used only for calculating *R*-factors(gt) *etc*. and is not relevant to the choice of reflections for refinement. *R*-factors based on *F*^2^ are statistically about twice as large as those based on *F*, and *R*- factors based on ALL data will be even larger.

### Fractional atomic coordinates and isotropic or equivalent isotropic displacement parameters (Å^2^)

**Table:**

	*x*	*y*	*z*	*U*_iso_*/*U*_eq_	
C1	0.8695 (5)	0.05079 (18)	0.3551 (3)	0.0601 (12)	
H1A	0.8702	0.0556	0.4205	0.090*	
H1B	0.9482	0.0180	0.3448	0.090*	
H1C	0.7601	0.0383	0.3255	0.090*	
C2	0.9159 (5)	0.11345 (19)	0.3160 (3)	0.0439 (9)	
C3	1.0243 (5)	0.1263 (2)	0.2541 (3)	0.0495 (10)	
H3	1.0856	0.0958	0.2271	0.059*	
C4	1.0250 (5)	0.19112 (19)	0.2401 (3)	0.0447 (9)	
C5	1.1171 (5)	0.2293 (2)	0.1789 (3)	0.0578 (11)	
H5A	1.2074	0.2519	0.2159	0.087*	
H5B	1.0431	0.2600	0.1444	0.087*	
H5C	1.1604	0.2006	0.1369	0.087*	
C6	0.8625 (4)	0.28073 (18)	0.3082 (2)	0.0384 (8)	
C7	0.9490 (5)	0.3351 (2)	0.2864 (3)	0.0484 (10)	
H7	1.0456	0.3310	0.2605	0.058*	
C8	0.8881 (5)	0.3949 (2)	0.3042 (3)	0.0518 (11)	
H8	0.9446	0.4319	0.2903	0.062*	
C9	0.7434 (5)	0.40128 (17)	0.3424 (2)	0.0430 (9)	
C10	0.6661 (5)	0.34335 (17)	0.3633 (2)	0.0377 (8)	
C11	0.5198 (5)	0.34594 (17)	0.4057 (2)	0.0367 (8)	
C12	0.4500 (5)	0.40613 (17)	0.4212 (2)	0.0411 (9)	
C13	0.5276 (5)	0.46377 (18)	0.3973 (3)	0.0500 (10)	
H13	0.4793	0.5037	0.4062	0.060*	
C14	0.6701 (5)	0.46183 (18)	0.3618 (3)	0.0509 (11)	
H14	0.7217	0.5004	0.3496	0.061*	
C15	0.3018 (5)	0.40495 (18)	0.4590 (3)	0.0465 (10)	
H15	0.2483	0.4437	0.4684	0.056*	
C16	0.2359 (5)	0.34780 (18)	0.4821 (3)	0.0453 (9)	
H16	0.1387	0.3472	0.5079	0.054*	
C17	0.3165 (4)	0.28978 (17)	0.4663 (2)	0.0356 (8)	
C18	0.0569 (5)	0.2532 (2)	0.6005 (3)	0.0634 (12)	
H18A	0.0072	0.2285	0.6445	0.095*	
H18B	−0.0289	0.2741	0.5578	0.095*	
H18C	0.1297	0.2855	0.6325	0.095*	
C19	0.1546 (5)	0.20902 (18)	0.5488 (3)	0.0418 (9)	
C20	0.1604 (5)	0.14376 (19)	0.5516 (3)	0.0484 (10)	
H20	0.1023	0.1166	0.5860	0.058*	
C21	0.2705 (5)	0.12476 (18)	0.4929 (3)	0.0467 (10)	
C22	0.3248 (6)	0.05741 (19)	0.4726 (3)	0.0691 (14)	
H22A	0.3806	0.0584	0.4196	0.104*	
H22B	0.2290	0.0296	0.4601	0.104*	
H22C	0.3995	0.0411	0.5252	0.104*	
C23	0.4455 (4)	0.10746 (17)	0.2012 (3)	0.0410 (9)	
C24	0.7410 (4)	0.12250 (18)	0.5941 (3)	0.0403 (9)	
Cd1	0.58715 (3)	0.191050 (12)	0.393278 (17)	0.03432 (10)	
N1	0.8486 (4)	0.16784 (15)	0.3401 (2)	0.0415 (7)	
N2	0.9165 (4)	0.21615 (15)	0.2943 (2)	0.0396 (7)	
N3	0.7267 (4)	0.28377 (14)	0.34580 (19)	0.0356 (7)	
N4	0.4537 (4)	0.28927 (14)	0.42863 (19)	0.0348 (7)	
N5	0.2597 (4)	0.22869 (13)	0.4890 (2)	0.0370 (7)	
N6	0.3316 (4)	0.17518 (14)	0.4562 (2)	0.0431 (8)	
N7	0.4794 (4)	0.13517 (17)	0.2698 (2)	0.0547 (9)	
N8	0.7010 (4)	0.14735 (18)	0.5244 (2)	0.0588 (10)	
S1	0.80382 (16)	0.08898 (6)	0.69384 (8)	0.0680 (4)	
S2	0.39440 (16)	0.07173 (7)	0.10205 (8)	0.0730 (4)	

### Atomic displacement parameters (Å^2^)

**Table:**

	*U*^11^	*U*^22^	*U*^33^	*U*^12^	*U*^13^	*U*^23^
C1	0.070 (3)	0.042 (2)	0.075 (3)	0.006 (2)	0.034 (3)	0.004 (2)
C2	0.040 (2)	0.047 (2)	0.046 (2)	0.0034 (18)	0.0100 (18)	0.0002 (19)
C3	0.044 (2)	0.052 (2)	0.057 (3)	0.0061 (19)	0.021 (2)	−0.001 (2)
C4	0.038 (2)	0.055 (2)	0.044 (2)	0.0012 (19)	0.0133 (17)	0.0005 (19)
C5	0.057 (3)	0.066 (3)	0.058 (3)	−0.001 (2)	0.034 (2)	0.007 (2)
C6	0.038 (2)	0.042 (2)	0.034 (2)	−0.0040 (17)	0.0021 (16)	0.0048 (17)
C7	0.043 (2)	0.052 (2)	0.051 (2)	−0.0088 (19)	0.0112 (19)	0.000 (2)
C8	0.055 (3)	0.044 (2)	0.055 (3)	−0.015 (2)	0.006 (2)	0.006 (2)
C9	0.052 (2)	0.037 (2)	0.037 (2)	−0.0069 (18)	0.0000 (18)	0.0010 (17)
C10	0.044 (2)	0.038 (2)	0.0288 (19)	−0.0029 (17)	−0.0004 (16)	−0.0012 (16)
C11	0.043 (2)	0.0364 (19)	0.0289 (19)	−0.0032 (17)	−0.0002 (15)	−0.0013 (16)
C12	0.056 (2)	0.0329 (19)	0.0306 (19)	0.0058 (18)	−0.0032 (17)	−0.0053 (16)
C13	0.070 (3)	0.034 (2)	0.043 (2)	0.004 (2)	0.000 (2)	−0.0053 (17)
C14	0.070 (3)	0.035 (2)	0.044 (2)	−0.007 (2)	−0.002 (2)	−0.0017 (18)
C15	0.056 (3)	0.037 (2)	0.046 (2)	0.0133 (19)	0.0030 (19)	−0.0034 (18)
C16	0.044 (2)	0.045 (2)	0.047 (2)	0.0158 (19)	0.0072 (18)	−0.0026 (19)
C17	0.038 (2)	0.0354 (19)	0.0329 (19)	0.0031 (16)	0.0046 (16)	−0.0022 (15)
C18	0.064 (3)	0.064 (3)	0.071 (3)	−0.002 (2)	0.036 (2)	−0.009 (2)
C19	0.035 (2)	0.047 (2)	0.045 (2)	0.0031 (17)	0.0114 (17)	−0.0026 (18)
C20	0.045 (2)	0.047 (2)	0.057 (3)	−0.0055 (19)	0.0202 (19)	0.004 (2)
C21	0.042 (2)	0.038 (2)	0.063 (3)	0.0004 (18)	0.0176 (19)	0.001 (2)
C22	0.068 (3)	0.038 (2)	0.112 (4)	0.002 (2)	0.046 (3)	0.000 (3)
C23	0.040 (2)	0.037 (2)	0.049 (2)	−0.0033 (17)	0.0154 (18)	0.0010 (18)
C24	0.037 (2)	0.046 (2)	0.040 (2)	0.0055 (17)	0.0137 (17)	−0.0028 (18)
Cd1	0.03855 (16)	0.03186 (15)	0.03399 (16)	0.00236 (12)	0.01020 (11)	0.00097 (11)
N1	0.0418 (18)	0.0401 (17)	0.0451 (19)	0.0025 (14)	0.0149 (15)	0.0044 (15)
N2	0.0342 (17)	0.0435 (17)	0.0423 (18)	−0.0006 (14)	0.0099 (14)	0.0014 (15)
N3	0.0417 (18)	0.0303 (14)	0.0357 (17)	−0.0002 (13)	0.0093 (14)	0.0073 (13)
N4	0.0357 (17)	0.0333 (15)	0.0359 (16)	0.0038 (13)	0.0072 (13)	0.0029 (13)
N5	0.0371 (17)	0.0341 (17)	0.0410 (17)	0.0039 (13)	0.0095 (13)	−0.0029 (14)
N6	0.0455 (19)	0.0345 (17)	0.054 (2)	0.0052 (14)	0.0209 (16)	−0.0020 (14)
N7	0.053 (2)	0.064 (2)	0.049 (2)	−0.0099 (18)	0.0115 (17)	−0.0110 (18)
N8	0.062 (2)	0.076 (3)	0.039 (2)	0.017 (2)	0.0101 (17)	0.0153 (19)
S1	0.0826 (9)	0.0803 (8)	0.0411 (6)	0.0306 (7)	0.0101 (6)	0.0166 (6)
S2	0.0951 (10)	0.0682 (8)	0.0584 (7)	−0.0295 (7)	0.0204 (7)	−0.0252 (6)

### Geometric parameters (Å, °)

**Table:**

C1—C2	1.486 (5)	C15—C16	1.359 (5)
C1—H1A	0.9600	C15—H15	0.9300
C1—H1B	0.9600	C16—C17	1.400 (5)
C1—H1C	0.9600	C16—H16	0.9300
C2—N1	1.320 (5)	C17—N4	1.323 (4)
C2—C3	1.388 (5)	C17—N5	1.399 (4)
C3—C4	1.351 (5)	C18—C19	1.493 (5)
C3—H3	0.9300	C18—H18A	0.9600
C4—N2	1.379 (5)	C18—H18B	0.9600
C4—C5	1.482 (5)	C18—H18C	0.9600
C5—H5A	0.9600	C19—C20	1.346 (5)
C5—H5B	0.9600	C19—N5	1.379 (5)
C5—H5C	0.9600	C20—C21	1.394 (5)
C6—N3	1.312 (5)	C20—H20	0.9300
C6—C7	1.388 (5)	C21—N6	1.304 (5)
C6—N2	1.426 (5)	C21—C22	1.500 (5)
C7—C8	1.368 (6)	C22—H22A	0.9600
C7—H7	0.9300	C22—H22B	0.9600
C8—C9	1.389 (6)	C22—H22C	0.9600
C8—H8	0.9300	C23—N7	1.149 (4)
C9—C10	1.406 (5)	C23—S2	1.620 (4)
C9—C14	1.431 (5)	C24—N8	1.140 (4)
C10—N3	1.362 (5)	C24—S1	1.621 (4)
C10—C11	1.430 (5)	Cd1—N8	2.185 (3)
C11—N4	1.350 (5)	Cd1—N7	2.201 (3)
C11—C12	1.398 (5)	Cd1—N3	2.382 (3)
C12—C15	1.406 (5)	Cd1—N4	2.392 (3)
C12—C13	1.415 (5)	Cd1—N1	2.428 (3)
C13—C14	1.345 (6)	Cd1—N6	2.428 (3)
C13—H13	0.9300	N1—N2	1.366 (4)
C14—H14	0.9300	N5—N6	1.371 (4)
			
C2—C1—H1A	109.5	C19—C18—H18B	109.5
C2—C1—H1B	109.5	H18A—C18—H18B	109.5
H1A—C1—H1B	109.5	C19—C18—H18C	109.5
C2—C1—H1C	109.5	H18A—C18—H18C	109.5
H1A—C1—H1C	109.5	H18B—C18—H18C	109.5
H1B—C1—H1C	109.5	C20—C19—N5	106.9 (3)
N1—C2—C3	110.3 (3)	C20—C19—C18	127.8 (4)
N1—C2—C1	119.5 (3)	N5—C19—C18	125.3 (3)
C3—C2—C1	130.2 (4)	C19—C20—C21	106.5 (4)
C4—C3—C2	107.8 (4)	C19—C20—H20	126.7
C4—C3—H3	126.1	C21—C20—H20	126.7
C2—C3—H3	126.1	N6—C21—C20	110.8 (3)
C3—C4—N2	105.3 (3)	N6—C21—C22	120.7 (4)
C3—C4—C5	129.1 (4)	C20—C21—C22	128.4 (4)
N2—C4—C5	125.6 (4)	C21—C22—H22A	109.5
C4—C5—H5A	109.5	C21—C22—H22B	109.5
C4—C5—H5B	109.5	H22A—C22—H22B	109.5
H5A—C5—H5B	109.5	C21—C22—H22C	109.5
C4—C5—H5C	109.5	H22A—C22—H22C	109.5
H5A—C5—H5C	109.5	H22B—C22—H22C	109.5
H5B—C5—H5C	109.5	N7—C23—S2	177.2 (4)
N3—C6—C7	123.4 (4)	N8—C24—S1	177.9 (4)
N3—C6—N2	113.9 (3)	N8—Cd1—N7	124.13 (14)
C7—C6—N2	122.7 (4)	N8—Cd1—N3	115.61 (12)
C8—C7—C6	118.1 (4)	N7—Cd1—N3	108.99 (11)
C8—C7—H7	121.0	N8—Cd1—N4	107.53 (11)
C6—C7—H7	121.0	N7—Cd1—N4	119.05 (11)
C7—C8—C9	121.2 (4)	N3—Cd1—N4	68.85 (11)
C7—C8—H8	119.4	N8—Cd1—N1	86.08 (12)
C9—C8—H8	119.4	N7—Cd1—N1	83.53 (12)
C8—C9—C10	116.4 (4)	N3—Cd1—N1	65.22 (10)
C8—C9—C14	124.8 (4)	N4—Cd1—N1	133.44 (10)
C10—C9—C14	118.8 (4)	N8—Cd1—N6	83.01 (12)
N3—C10—C9	122.4 (3)	N7—Cd1—N6	89.38 (12)
N3—C10—C11	117.8 (3)	N3—Cd1—N6	134.12 (10)
C9—C10—C11	119.7 (3)	N4—Cd1—N6	65.52 (10)
N4—C11—C12	122.6 (4)	N1—Cd1—N6	160.61 (11)
N4—C11—C10	117.9 (3)	C2—N1—N2	105.6 (3)
C12—C11—C10	119.5 (3)	C2—N1—Cd1	132.5 (3)
C11—C12—C15	116.4 (3)	N2—N1—Cd1	117.3 (2)
C11—C12—C13	119.7 (4)	N1—N2—C4	111.0 (3)
C15—C12—C13	123.9 (4)	N1—N2—C6	116.8 (3)
C14—C13—C12	121.2 (4)	C4—N2—C6	132.2 (3)
C14—C13—H13	119.4	C6—N3—C10	118.5 (3)
C12—C13—H13	119.4	C6—N3—Cd1	123.8 (2)
C13—C14—C9	121.0 (4)	C10—N3—Cd1	117.6 (2)
C13—C14—H14	119.5	C17—N4—C11	119.6 (3)
C9—C14—H14	119.5	C17—N4—Cd1	122.7 (2)
C16—C15—C12	120.7 (3)	C11—N4—Cd1	117.7 (2)
C16—C15—H15	119.6	N6—N5—C19	109.2 (3)
C12—C15—H15	119.6	N6—N5—C17	117.7 (3)
C15—C16—C17	119.0 (4)	C19—N5—C17	132.6 (3)
C15—C16—H16	120.5	C21—N6—N5	106.5 (3)
C17—C16—H16	120.5	C21—N6—Cd1	132.1 (2)
N4—C17—N5	115.2 (3)	N5—N6—Cd1	117.7 (2)
N4—C17—C16	121.6 (4)	C23—N7—Cd1	169.8 (3)
N5—C17—C16	123.2 (3)	C24—N8—Cd1	171.6 (3)
C19—C18—H18A	109.5		
			
N1—C2—C3—C4	0.5 (5)	C11—C10—N3—Cd1	−2.2 (4)
C1—C2—C3—C4	−179.7 (4)	N8—Cd1—N3—C6	77.8 (3)
C2—C3—C4—N2	0.1 (5)	N7—Cd1—N3—C6	−67.6 (3)
C2—C3—C4—C5	−178.7 (4)	N4—Cd1—N3—C6	177.7 (3)
N3—C6—C7—C8	−0.8 (6)	N1—Cd1—N3—C6	5.6 (3)
N2—C6—C7—C8	−178.8 (3)	N6—Cd1—N3—C6	−176.1 (2)
C6—C7—C8—C9	−0.3 (6)	N8—Cd1—N3—C10	−98.7 (2)
C7—C8—C9—C10	1.2 (6)	N7—Cd1—N3—C10	115.9 (2)
C7—C8—C9—C14	−179.0 (4)	N4—Cd1—N3—C10	1.3 (2)
C8—C9—C10—N3	−1.1 (5)	N1—Cd1—N3—C10	−170.9 (3)
C14—C9—C10—N3	179.1 (3)	N6—Cd1—N3—C10	7.4 (3)
C8—C9—C10—C11	177.9 (3)	N5—C17—N4—C11	−178.9 (3)
C14—C9—C10—C11	−1.9 (5)	C16—C17—N4—C11	0.9 (5)
N3—C10—C11—N4	2.1 (5)	N5—C17—N4—Cd1	3.4 (4)
C9—C10—C11—N4	−177.0 (3)	C16—C17—N4—Cd1	−176.7 (2)
N3—C10—C11—C12	−177.3 (3)	C12—C11—N4—C17	0.8 (5)
C9—C10—C11—C12	3.6 (5)	C10—C11—N4—C17	−178.7 (3)
N4—C11—C12—C15	−2.4 (5)	C12—C11—N4—Cd1	178.5 (2)
C10—C11—C12—C15	177.0 (3)	C10—C11—N4—Cd1	−0.9 (4)
N4—C11—C12—C13	178.7 (3)	N8—Cd1—N4—C17	−71.1 (3)
C10—C11—C12—C13	−1.9 (5)	N7—Cd1—N4—C17	76.9 (3)
C11—C12—C13—C14	−1.6 (6)	N3—Cd1—N4—C17	177.5 (3)
C15—C12—C13—C14	179.6 (4)	N1—Cd1—N4—C17	−172.7 (2)
C12—C13—C14—C9	3.3 (6)	N6—Cd1—N4—C17	2.4 (3)
C8—C9—C14—C13	178.7 (4)	N8—Cd1—N4—C11	111.2 (2)
C10—C9—C14—C13	−1.6 (6)	N7—Cd1—N4—C11	−100.8 (2)
C11—C12—C15—C16	2.4 (5)	N3—Cd1—N4—C11	−0.2 (2)
C13—C12—C15—C16	−178.7 (3)	N1—Cd1—N4—C11	9.6 (3)
C12—C15—C16—C17	−0.9 (5)	N6—Cd1—N4—C11	−175.3 (3)
C15—C16—C17—N4	−0.8 (5)	C20—C19—N5—N6	−0.8 (4)
C15—C16—C17—N5	179.0 (3)	C18—C19—N5—N6	178.3 (4)
N5—C19—C20—C21	0.1 (4)	C20—C19—N5—C17	−172.4 (3)
C18—C19—C20—C21	−179.0 (4)	C18—C19—N5—C17	6.7 (7)
C19—C20—C21—N6	0.7 (5)	N4—C17—N5—N6	−11.0 (4)
C19—C20—C21—C22	178.4 (4)	C16—C17—N5—N6	169.1 (3)
C3—C2—N1—N2	−0.8 (4)	N4—C17—N5—C19	160.0 (4)
C1—C2—N1—N2	179.3 (3)	C16—C17—N5—C19	−19.9 (6)
C3—C2—N1—Cd1	153.5 (3)	C20—C21—N6—N5	−1.2 (5)
C1—C2—N1—Cd1	−26.3 (5)	C22—C21—N6—N5	−179.2 (4)
N8—Cd1—N1—C2	74.0 (4)	C20—C21—N6—Cd1	155.8 (3)
N7—Cd1—N1—C2	−51.0 (3)	C22—C21—N6—Cd1	−22.2 (6)
N3—Cd1—N1—C2	−165.4 (4)	C19—N5—N6—C21	1.3 (4)
N4—Cd1—N1—C2	−175.5 (3)	C17—N5—N6—C21	174.3 (3)
N6—Cd1—N1—C2	18.2 (5)	C19—N5—N6—Cd1	−159.6 (2)
N8—Cd1—N1—N2	−134.0 (3)	C17—N5—N6—Cd1	13.4 (4)
N7—Cd1—N1—N2	101.0 (3)	N8—Cd1—N6—C21	−50.1 (4)
N3—Cd1—N1—N2	−13.4 (2)	N7—Cd1—N6—C21	74.4 (4)
N4—Cd1—N1—N2	−23.5 (3)	N3—Cd1—N6—C21	−169.3 (3)
N6—Cd1—N1—N2	170.2 (3)	N4—Cd1—N6—C21	−163.0 (4)
C2—N1—N2—C4	0.9 (4)	N1—Cd1—N6—C21	6.1 (6)
Cd1—N1—N2—C4	−158.1 (2)	N8—Cd1—N6—N5	104.8 (3)
C2—N1—N2—C6	179.5 (3)	N7—Cd1—N6—N5	−130.7 (3)
Cd1—N1—N2—C6	20.6 (4)	N3—Cd1—N6—N5	−14.4 (3)
C3—C4—N2—N1	−0.6 (4)	N4—Cd1—N6—N5	−8.1 (2)
C5—C4—N2—N1	178.2 (4)	N1—Cd1—N6—N5	161.1 (3)
C3—C4—N2—C6	−179.0 (3)	S2—C23—N7—Cd1	−89 (8)
C5—C4—N2—C6	−0.1 (6)	N8—Cd1—N7—C23	−97.1 (19)
N3—C6—N2—N1	−15.2 (4)	N3—Cd1—N7—C23	44.7 (19)
C7—C6—N2—N1	163.0 (3)	N4—Cd1—N7—C23	120.5 (19)
N3—C6—N2—C4	163.0 (4)	N1—Cd1—N7—C23	−16.3 (19)
C7—C6—N2—C4	−18.8 (6)	N6—Cd1—N7—C23	−178.2 (19)
C7—C6—N3—C10	0.9 (5)	S1—C24—N8—Cd1	−151 (9)
N2—C6—N3—C10	179.1 (3)	N7—Cd1—N8—C24	−72 (2)
C7—C6—N3—Cd1	−175.6 (3)	N3—Cd1—N8—C24	149 (2)
N2—C6—N3—Cd1	2.6 (4)	N4—Cd1—N8—C24	74 (2)
C9—C10—N3—C6	0.1 (5)	N1—Cd1—N8—C24	−151 (2)
C11—C10—N3—C6	−178.9 (3)	N6—Cd1—N8—C24	13 (2)
C9—C10—N3—Cd1	176.8 (2)		
